# Associations Between Dietary Patterns and Neuroimaging Markers: A Systematic Review

**DOI:** 10.3389/fnut.2022.806006

**Published:** 2022-04-26

**Authors:** Rebecca F. Townsend, Jayne V. Woodside, Federica Prinelli, Roisin F. O’Neill, Claire T. McEvoy

**Affiliations:** ^1^Centre for Public Health, Queen’s University Belfast, Belfast, United Kingdom; ^2^Institute for Global Food Security, Queen’s University Belfast, Belfast, United Kingdom; ^3^Institute of Biomedical Technologies, National Research Council, Milan, Italy

**Keywords:** dietary patterns, neuroimaging, neurocognition, diet, brain health

## Abstract

Dementia is a complex, growing challenge for population health worldwide. Dietary patterns (DPs) may offer an opportunity to beneficially influence cognitive ageing and potentially reduce an individuals’ risk of dementia through diet-related mechanisms. However, previous studies within this area have shown mixed results, which may be partly explained by the lack of sensitivity and accuracy within cognitive testing methods. Novel neuroimaging techniques provide a sensitive method to analyse brain changes preceding cognitive impairment which may have previously remained undetected. The purpose of this systematic review was to elucidate the role of DPs in relation to brain ageing processes, by summarising current prospective and intervention studies. Nine prospective studies met the inclusion criteria for the review, seven evaluated the Mediterranean diet (MeDi), one evaluated the Alternative Healthy Eating Index-2010, and one evaluated *a posteriori* derived DPs. No intervention studies were eligible for inclusion in this review. There was some evidence of an association between healthy DPs and neuroimaging markers including changes within these markers over time. Consequently, it is plausible that better adherence to such DPs may positively influence brain ageing and neurodegeneration. Future studies may benefit from the use of multi-modal neuroimaging techniques, to further investigate how adherence to a DP influences brain health. The review also highlights the crucial need for further intervention studies within this research area.

## Introduction

Dementia is a global challenge within the twenty-first century. Over 50 million individuals worldwide are estimated to live with the condition, a number that is expected to treble by 2050 (1). Up to 40% of future cases could be prevented or delayed by targeting modifiable factors (2). Previous observational research suggests greater adherence towards a healthy dietary pattern (DP) is associated with slower cognitive decline (3–7) and reduced risk of Alzheimer’s disease (AD) (the most common cause of dementia) (8–12) in later life. Yet, the limited data from randomised controlled trials (RCT) have not convincingly demonstrated a protective effect of diet on cognition. For example, two RCT’s found intervention with an enhanced Mediterranean diet (MeDi) enhanced with either nuts or extra-virgin olive oil (EVOO) improved Mini-Mental State Examination (MMSE) scores ranging from 4 to 6 years post-intervention, in older adults with high vascular risk (13, 14). However, a sub-study from the same cohort also found cognitive performance only improved significantly in those supplementing the MeDi with EVOO and not those that supplemented with nuts, when compared to controls (15). Similarly, another study also found intervention with a MeDi supplemented with dairy foods did not significantly improve cognition in middle-aged adults (16). Intervention studies investigating DPs other than the MeDi have also found inconsistent results (17–20).

A potential reason for the inconsistent findings may be the relatively short trial duration, making it difficult to discern effects on cognition and the narrow range of cognitive endpoints assessed. Subsequently, there is a need to determine which aspects of cognition are sensitive to dietary change, and the optimum timescale over which to observe these effects during trials. Furthermore, diet intervention is likely to have the most benefit during preclinical stages of AD, where it is more difficult to detect such minimal deterioration in cognition. However, due to advancements in neuroimaging techniques, there is now the ability to sensitively measure changes within brain health, which can help to elucidate potential diet-related mechanisms of neurodegeneration. For example, accumulating cross-sectional data suggest a protective association of healthy DPs on brain structures and functions. In particular, adherence to the MeDi has been associated with reduced brain atrophy, specifically in AD-vulnerable regions (21) including the hippocampus (22) and posterior cingulate cortex (23). Furthermore, better adherence to the MeDi has also been associated with less amyloid-β (Aβ) burden (24).

There have been limited comprehensive reviews of whole DPs and neuroimaging biomarkers (25–27). A previous systematic review (28) evaluated evidence published up to 2017. However, several prospective studies investigating DPs and neuroimaging outcomes have been published since 2017, which have not yet been synthesized. More recently, a systematic review (25) suggested evidence of associations between healthy DPs and brain imaging correlates, which preceded cognitive decline. However, only studies reporting both cognitive performance and neuroimaging biomarkers were reviewed, meaning that several studies reporting neuroimaging markers alone were not eligible for review. A further review (26) investigated DPs in relation to neuroimaging markers but only in middle-aged adults, meaning studies including both younger and older age adults were excluded. An additional review (27), investigated any aspect of diet or metabolism in relation to neuroimaging markers and included some, but not all available (29, 30) studies investigating DPs in relation to neuroimaging markers. It should be noted that these latter three reviews included evidence from cross-sectional studies, from which a causal relationship between diet and neurocognitive outcomes cannot be established. Therefore, we aimed to synthesise the available data from RCTs and prospective studies to evaluate effects of DPs on neuroimaging biomarkers. This review will address the following research questions; (1) What is the relationship between DPs and neuroimaging markers across the adult life course? and (2) What is the effect of DPs on changes in neuroimaging measures over time?

## Methods

### Study Design and Systematic Review Protocol

This systematic review was undertaken based on the Centre for Reviews and Dissemination (CRD) guidance for undertaking systematic reviews in health care. The protocol for this review was registered with PROSPERO database (PROSPERO 2020: CRD42020181423). The review presented here focuses on neuroimaging outcomes from part of a broader review on DPs and neurocognitive outcomes.

### Search Strategy and Data Sources

A systematic literature search was conducted using two major databases; EMBASE and Ovid MEDLINE (a subset of PubMed), for relevant studies published up to 5 March 2020. A detailed search strategy (shown in Supplementary Table 1) was developed (28, 31), using key terms associated with DPs and neuroimaging markers. The primary search was limited to humans and English language publications. Articles were considered eligible for review if they were an RCT or prospective observational design and met the following criteria; (i) measured adherence/exposure to ≥1 whole DP, (ii) reported neuroimaging outcomes using any imaging measure/technique; (iii) evaluated the effect of diet in relation to any specified brain ageing outcome(s) or determined the association between diet and brain ageing outcomes. Intervention studies were eligible for inclusion if they were of RCT design and met the above criteria. We placed no restrictions on intervention duration or type. As studies were required to include ≥1 whole DP, those which focused on a single food, macronutrient group, were based on nutrients alone or focused on calorie restriction or weight loss, without overall consideration for dietary quality were excluded. Multi-domain lifestyle exposures/interventions were also excluded, due to the difficulty in disentangling potential effects from diet alone. Studies within the same cohort were excluded if the exposures and outcomes studied were analysed identically within each.

### Data Extraction

The process of study selection and data extraction was managed using EndNote software version X9 and Rayyan online software. Titles of all potentially eligible studies were screened by R.F.T. and those not including any keywords relating to the review were excluded. Two reviewers (R.F.T. and C.T.ME.) screened the abstracts of all remaining studies independently before assessing the remaining studies further for eligibility via retrieval and independent reading of full texts. Any discrepancies or queries regarding inclusion of studies were then resolved through discussion among the research team (R.F.T, C.T.ME., and J.V.W). For duplicated study cohorts reporting identical diet exposure and neuroimaging outcome(s) at different time points, the study with the longest follow-up period was included. Data was collected from each study identified as eligible for inclusion using a standardized form. For prospective studies included, the following information was collected; names of authors, year of publication and study cohort name (if applicable); country of study location; follow-up period (years); exclusion and inclusion criteria; method to derive DP (*a priori* or *a posteriori*); dietary assessment tools used (e.g., 24 h recall, food frequency questionnaire (FFQ), diet record, diet history); DP(s) examined; time points at which DP was examined; neuroimaging methods; primary outcomes effect size and/or summary of main findings; covariates included in adjusted models (where applicable).

### Risk of Bias Assessment

Two reviewers (R.F.T and R.F.ON.) independently examined the quality of each study included in the review using the Newcastle-Ottawa Scale (NOS) (32) for prospective studies. Studies could be awarded a maximum of nine stars in total. Up to four stars could be awarded for selection of study (representation within sample, selection of sample, exposure measurement, and demonstration outcome not present at baseline). Up to two stars could be awarded within the comparability domain (controls for basic confounders or includes additional confounding factors) comparability and up to three stars for outcome measurement (including methodology of outcome assessment, follow-up length, and adequacy of follow-up cohort). Studies were considered high quality if they scored 9 stars, medium quality if 7–8 stars were scored, or low quality if ≤6 stars were scored.

### Data Analysis

Due to the heterogeneity within the data collected in terms of neuroimaging markers, statistical analyses and reporting of data, a quantitative analysis was not possible. Consequently, a narrative synthesis was used to present results alongside detailed tables.

## Results

### Study Selection

**FIGURE 1:**
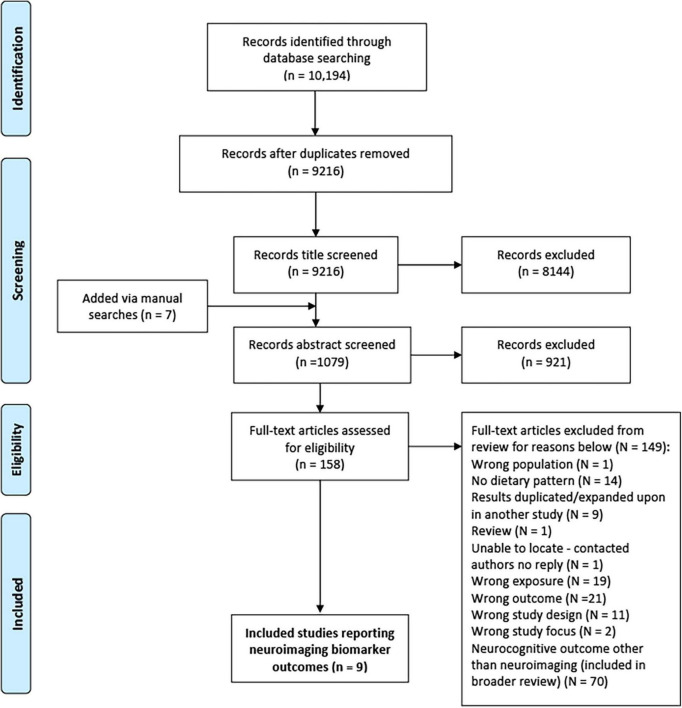
PRISMA flow diagram for process of study selection (87).

The preferred reporting items for systematic reviews and meta-analyses (PRISMA) flow diagram is shown in Figure 1. From the primary systematic search in March 2020, 10,194 articles were identified, 978 of which were removed due to duplication. Following title and abstract screening, 9,065 articles were excluded. From the remaining 158 full text articles, a further 149 were excluded (see Figure 1). A total of 79 articles [70 prospective studies and 9 randomised control trials (RCT)] were therefore included within the broader systematic review; 9 of which included neuroimaging markers and were included in this review.

### Study Characteristics

All included articles were prospective studies published in the last 10 years (29, 30, 33–39). There were no RCTs identified. Sample sizes varied between studies ranging from 70 to 707, with a total of approximately 2,540 participants across all articles. Similarly, follow-up periods also differed greatly with the shortest period being 3 years to the longest period of 13 years. An overview of the key characteristics and findings of each study included within the review is provided in Table 1.

**TABLE 1:** Overview of included studies investigating DPs in relation to neuroimaging markers.

Authors, year, study name, country	Study characteristics			Diet measures			Neuroimaging measures	Results			
	Follow-up time (years)	N=	Population characteristics	Mean age at baseline, y	Food intake assessment; time point(s) assessed	DP(s) examined (scoring reference or *a posteriori*)	Specific methods/ techniques used:	Timepoint(s) of neuroimaging assessment(s)	Primary outcome(s) of interest	Main findings from primary outcome(s) of interest	Covariates
Pelletier et al. (29); *Three City Study;* France.	8.9	146	Adults ≥ 65 years who are non-institutionalised free from dementia at baseline.	73	40-item FFQ and 24-h diet recall, administered by trained dietitian; 1 time point used in analysis, but reassessments conducted to assess diet stability in supplementary analyses.	1. Mediterranean diet, Trichopoulou et al. (40)	3 T MRI and DTI.	1 time point; 8.9 years on average post-diet assessment.	GMV, WMV, WM microstructure via fractional anisotropy, axial diffusivity, radial diffusivity and mean diffusivity (global measure of diffusion).	Adherence to MeDi was not significantly associated with any structural MRI measures (GMV, WMV and TIV) but better MeDi adherence was associated with reduced mean diffusivity values and higher fractional anisotropy values assessed 8.9 years later.	Age, sex, education, APOE4 caloric intake, BMI, smoking, physical activity, vascular risk factors (including cardiovascular or cerebrovascular disease, hypertension, diabetes and hypercholesterolem ia), cognitive performance on Isaac’s set test.
Scarmeas et al. (37); WHICAP Study; USA.	5.8 ± 3.22	707	Adults ≥ 65 years who are non-institutionalised living in Manhattan from WHICAP 1992 and 1999. 66% female	80.3 ± 5.7	Interviewer administered 61-item Willett Semi-quantitative FFQ; 1 time point (baseline).	1. Mediterranean diet, Trichopoulou et al. (40)	1.5 T MRI	1 time point; 5.8 years after baseline.	Cerebral infarcts and WMH.	Adherence to MeDi was not significantly associated with WMH but was associated with reduced odds of cerebral infarcts assessed 5.8 years later. Significance was attenuated after excluding individuals with dementia and stroke.	Age, sex, ethnicity, education, APOE E4 status, caloric intake, BMI, duration between diet evaluation and MRI, smoking, diabetes, hypertension, and heart disease.
Titova et al. (38); *Prospective Investigation of the Vasculature in Uppsala Seniors Cohort;* Sweden.	5	194	Adults ≥ 70 years who are community-dwelling and mostly clinically and cognitively normal at baseline. 48% female	70.1 ± 0.01	7-day food diary; 1 time point (baseline).	1. Mediterranean diet, Trichopoulou et al. (40)	1.5 T MRI	1 time point; 5 years after baseline.	GMV, WMV, TBV (sum of GMV and WMV).	Adherence to MeDi was not significantly associated with any structural MRI measures (GMV, WMV or TBV) assessed 5 years later.	Age, gender, education, caloric intake, BMI, physical activity, systolic blood pressure, HOMA-IR, LDL cholesterol.
Berti et al. (34), USA.	3	70	Participants derived from multiple community sources. Must been aged 30-60 years at enrolment with ≥ 12 years of education. MeDi- group = 54% female MeDi + group = 64% female	MeDi- group = 50 ± 9; MeDi + group = 49 ± 9	Interviewer administered Willett Semi-quantitative FFQ; 1 time point.	1. Mediterranean diet, Trichopoulou et al. (40)	3 T MRI, PiB-PET and FDG-PET	2 time points; baseline and ≥ 2 years follow-up.	GMV, 11C Pittsburgh compound-B (PiB) uptake (a known marker of fibrillary amyloid-β) and 18-F fluorodeoxyglucose (FDG) (a known marker of glucose metabolism)-PET.	Adherence to MeDi was not significantly associated with GMV or change in GMV across 2 years. However lower adherence was significantly associated with increased CMRglc decline and increased Aβ load.	Age, sex, education, APOE E4 status. BMI, insulin resistance, hypertension.
Walters et al. (39), USA.	3	70	Cognitively and clinically normal middle-aged adults, age 30–60 years at baseline.	49 ± 8	Willett Semi-quantitative FFQ; 1 time point.	1. Mediterranean diet, Trichopoulou et al. (40)	3 T MRI, PiB-PET and FDG-PET	2 time points; baseline and ≥ 2 years follow-up.	Entorhinal and posterior cingulate cortices thickness, PiB uptake and FDG of posterior cingulate and frontal cortices.	Adherence to MeDi was not significantly associated with cortices thickness or PiB uptake at baseline or 2 year follow up. Lower adherence to MeDi was significantly associated with increased rate of FDG decline in posterior cingulate cortex but not in frontal cortex.	Age, sex, education, BMI, APOE E4 status, diet, physical activity, hypertension and intellectual scores, QUICKI scores, lab measures.
Luciano et al. (36), *Lothian Birth Cohort*, Scotland, UK.	3	562/401	Adults born in 1936 who are community dwelling and free from dementia at baseline. 47.9% female	72.65 ± 0.72	168-item FFQ; 1 time point (baseline).	1. Mediterranean diet, Trichopoulou et al. (40)	1.5 T MRI.	2 time points; wave 2 (2007–2010) and wave 3 (2011–2014).	GMV, and Total Brain Volume and (representing difference in volume of CSF, venous sinuses and meninges and ICV), cortical thickness.	Adherence to MeDi was not significantly associated with GMV or cortical thickness at baseline or change across 3 years. Increased adherence to MeDi was significantly associated with reduced 3 year change in TBV but not with TBV at baseline or follow-up.	Age, sex, education, BMI, APOE E4 status, diabetes, stroke, blood pressure, cardiovascular disease, cognitive ability and MMSE.
Rainey-Smith et al. (30), *Australian Imaging, Biomarkers and Lifestyle Study of Ageing*, Australia.	3	77	Healthy, cognitively normal individuals aged 60 or above residing within Australia (analysis only completed on individuals considered as “Aβ accumulators”). 49% female	71.1 ± 7.1	74-item FFQ; 1 time point (baseline).	1. Mediterranean diet, Trichopoulou et al. (40)	PiB-PET	3 time points; Baseline, 18 month follow-up and 36 month follow-up.	Cerebral Aβ load.	Increased adherence to MeDi was significantly associated with decreased Aβ accumulation over 3 years.	Age, gender, education, caloric intake, BMI, APOE E4 status.
Akbaraly et al. (33), *Whitehall II Imaging Sub-study;* UK.	13	459	Civil servants from London aged 35–55 at baseline of Whitehall II Study. Aged 60–85 at baseline of imaging sub-study. 19.2% female	59.6 ± 5.3	127-item semi-quantitative FFQ; 3 time point(s) across 11 year period.	1. AHEI-2010, Chiuve et al. (41).	3 T MRI	1 time point; 13 years following baseline diet assessment.	Total hippocampal volume (HCV); Left HCV and right HCV.	Better AHEI-2010 adherence was associated with larger total HCV, left HCV and right HCV. Compared to those who maintained a low AHEI-2010 score over 11 year follow up, those maintaining a high score or improving their score to high had larger hippocampal volumes.	Age, sex, caloric intake, ethnicity, occupational position, smoking, physical activity, cardio-metabolic health factors (CHD, dyslipidaemia, type 2 diabetes, BMI and hypertension), cognitive impairment, depressive symptoms.
Jacka et al. (35), *PATH Sub-study*, Australia.	4	255	Oldest cohort of the PATH study (60–64 years old at baseline). 46% female	62.6 ± 1.42	183-item FFQ; 1 time point (baseline).	1. Prudent diet, derived *a posteriori*. 2. Western diet, derived *a posteriori*.	1.5 T MRI.	2 time points; baseline and follow-up at 3–4 years.	Total HCV, left HCV, right HCV and amygdala volumes.	Increased adherence to prudent DP was significantly associated with larger left HCV but not right HCV. Whereas, increased adherence to western DP was significantly associated with smaller left HCV but not right HCV. No significant association was found between either DP and 4 year change HCV.	Age, sex, education, labour-force status, physical activity, smoking, hypertension, diabetes, depressive symptoms, medication, intracranial volume, time between MRI and change in intracranial volume over time.

#### Populations Assessed

Most studies (6/9) included older aged adults (aged ≥ 60 years) (29, 30, 33, 35–38). Only two studies specifically included individuals from both young adulthood (age ≥ 30 years) and older adulthood (aged ≥ 60 years) (34, 39). Three studies were conducted in the United States of America (USA) (34, 37, 39), two from the United Kingdom (UK) (33, 36), two from Australia (30, 35), one from France (29), and one from Sweden (38).

All studies except for one (33) specified that at study baseline, participants must have been cognitively healthy and/or free from dementia. Although, one study did specifically select individuals categorised as Aβ accumulators (30). However, at the time of neuroimaging measurement, three studies reported the inclusion of individuals with cognitive impairment and/or a diagnosis of dementia (33, 37, 38). Two of these studies reported sensitivity analyses excluding those with cognitive impairment (33, 37).

The majority of studies were conducted in educated populations, with three specifically requiring ≥12 years of education to participate in the study (30, 34, 39).

Another demographic factor that may impact the relationship between DP and neuroimaging measures is ethnicity. Populations such as Black and Asian ethnic groups may experience a higher risk of developing AD (2). Yet, only four of nine included studies specifically reported participants’ ethnic demographics. Three studies (33, 34, 39) reported a high prevalence (≥70%) of white participants. In contrast, the proportion of white individuals within the Washington Heights-Hamilton Heights-Inwood Community Aging Project (WHICAP) study (37) (27%) was lower than African American individuals (35%) and Hispanic individuals (36%).

#### Measurement of Dietary Patterns

##### Assessment of Dietary Intake

Food frequency questionnaires were used in the majority of studies (8/9) to assess dietary intake. Of which, one study combined the FFQ used with another method, which was a 24-h dietary recall (29). The nature of FFQs means they lend themselves to self-reporting. However, from the studies within this review, three specifically reported that a trained individual (i.e., a dietitian or interviewer, respectively) administered the FFQ (29, 34, 37). In the one study that did not use an FFQ, a 7-day food diary was used (38).

##### Time Points of Diet Intake Assessment

Dietary intake data was captured at a single time point at baseline in nearly all studies (8/9) to measure adherence to the DPs studied in relation to neuroimaging markers (29, 30, 34–39). One study (33) evaluated diet exclusively retrospectively, to determine earlier life diet exposure. Diet measures were then repeated to evaluate a cumulative average and provide a value representative of longer-term adherence to the DP. It should be noted that one other study (29) also assessed diet stability through repeated diet measures within supplementary analyses, but not within main statistical analyses.

##### Dietary Pattern Indices

*A priori* scoring indices were used in eight studies to determine adherence towards a DP (29, 30, 33–39). One study utilised *a posteriori* methods (35). Details of the DPs studied within each article, including their respective scoring systems and food components are summarised in Supplementary Table 2.

Of the eight studies investigating *a priori* derived DPs, seven (29, 30, 34, 36–39) examined the MeDi and one (33) examined the Alternate Healthy Eating Index-2010 (AHEI-2010). Within the seven studies investigating the MeDi, all were based on the scoring index from Trichopoulou et al. (40), which is based on population median cut points for nine specific food components. Of which, five measured adherence to the MeDi as a continuous variable (29, 30, 36, 38, 39), one as a categorical variable (34), and one used both (37). The one study (33) which examined the AHEI-2010 used an adaptation of the index from Chiuve et al. (41). Adherence to the AHEI-2010 was measured as a continuous variable in main analyses and as a categorical variable in further analyses.

Only one study used *a posteriori* methodology in the form of principal component analysis (PCA). In contrast to *a priori* methodology, this adopts an exploratory approach and relies on dietary intake information provided to identify correlated items to enable the construction of common consumption patterns (DPs). The study included within this review defined the two DPs identified as prudent and western, which can be dichotomised as healthy and unhealthy, respectively (35). In regards to the management of energy misreporting from FFQs, some studies (30, 36, 38) reported using methods to address this.

#### Neuroimaging Measures

Magnetic resonance imaging (MRI) was used in eight studies to measure neuroimaging markers. MRI alone was used in five studies (33, 35–38), and in combination with either diffusion tensor imaging (DTI) (29) or positron emission tomography (PET) in three studies (34, 39). Neuroimaging markers were assessed at one time point in four studies (29, 30, 33, 36–38), two time points in four other studies (34–36, 39), and three time points in one study (30).

### Results of Main Outcome Analyses

A graphical overview of each of the DPs studied in relation to either their positive or null association to neuroimaging markers is provided within Table 2.

**TABLE 2:** Graphical overview of each of the DPs studied in relation to their association to neuroimaging markers.

Structural neuroimaging markers assessed in relation to MeDi					
Grey matter volume (GMV)		(29)	(38)	(34)	(36)
White matter volume (WMV)		(29)	(38)		
Total brain volume (TBV)		(38)	(36)		
Cortical thickness		(39)	(36)		
White matter hyperintensities (WMH)		(37)			
Cerebral infarcts		(37)			
White matter integrity (via DTI)		(29)			
**Functional neuroimaging markers assessed in relation to MeDi**					
Glucose metabolism (via FDG-PET)		(34)	(39)		
Aβ load (via PiB-PET)		(34)	(39)	(30)	
**Structural neuroimaging markers assessed in relation to other DPs**					
Hippocampal volume		(33)	(35)		
**Colour codes:**					
Positive association with neuroimaging markers measured at one time point.	Positive association with neuroimaging markers measured at two or more time points.				
No association with neuroimaging markers measured at one time point.	No association with neuroimaging markers measured at two or more time points.				

#### Mediterranean Diet and Structural Neuroimaging Markers

Six studies assessed the relationship between MeDi adherence and structural neuroimaging markers. These include grey matter volume (GMV), total brain volume (TBV), cortical thickness, white matter volume (WMV), white matter integrity (WMI), white matter hyperintensities (WMH) and cerebral infarcts.

##### Grey Matter Volumes

In total, four studies (29, 34, 36, 38) analysed the association between GMV and adherence to the MeDi. Two studies found no significant association between adherence to MeDi and GMV measured between 5 and 8.9 years later (29, 38). Two other studies (34, 36) included repeated measures of GMV. In the Lothian birth cohort, no significant association was found between adherence to the MeDi and GMV at baseline (β = 0.182; *SE* = 0.553; *P* = 0.742), follow-up (β = 0.864; *SE* = 0.659; *P* = 0.191), or change in GMV longitudinally across 3 years in older cognitively healthy adults (β = 0.451; *SE* = 0.383; *P* = 0.240) (36). Similarly, Berti and colleagues also found no significant differences between the high and low MeDi adherence and change in GMV over 3 years in middle-aged adults from the USA (34).

##### Total Brain Volumes

Two studies (36, 38) assessed TBV. One study (38) found MeDi adherence was not significantly associated with TBV measured at one time point 5 years following baseline. The other study (36) found no associations between MeDi adherence and TBV at either baseline or follow-up among older cognitively healthy adults, but lower MeDi adherence was significantly associated with greater reductions in TBV across 3 years (β = 0.976; *SE* = 0.483; *P* = 0.044) (36).

##### Cortical Thickness

Two prospective studies examined cortical thickness in relation to MeDi adherence (36, 39). In the Lothian birth cohort, MeDi adherence was not related to 3-year change in cortical thickness between 73 and 76 years (β = 0.004; *SE* = 0.003; *P* = 0.198) (36). Likewise, Walters and colleagues also found no significant association between adherence to the MeDi and thickness of the frontal cortex or the posterior cingulate cortex at baseline or follow-up (39). Nor was there a significant association between 2-year change in frontal or posterior cingulate cortical thickness and MeDi adherence (39).

##### White Matter Volumes

Two studies determined WMV in relation to MeDi adherence (29, 38). Neither found an association between MeDi adherence and WMV measured at an average of 5 or 8.9 years later (29, 38).

##### White Matter Integrity

Only one study (29) assessed WMI. The study found a significant association between increased adherence to the MeDi and reduced diffusivity values within specific areas of the white matter skeleton (the whole corpus callosum, anterior and posterior thalamic radiations, para cingulate gyrus cingulum, and parahippocampal fornix) among older French adults (≥65 years). Better adherence to the MeDi was also associated with increased fractional anisotropy values in specific regions of the white matter skeleton (corpus callosum, anterior and posterior thalamic radiations) (29). Authors consequently explored further the associations between diffusivity and fractional anisotropy values and cognitive tests. As a result, significant associations were found between higher global cognitive scores and the DTI parameters significantly associated with the MeDi (Multivariable adjusted mean difference when comparing Q1 to Q5 of mean diffusivity = 0.35; 95% CI: 0.06, 0.65; P for trend = 0.002). Higher global cognitive scores were also significantly associated with the DTI parameters significantly associated with the MeDi when comparing individuals in quartile one to quartile five of fractional anisotropy values (Multivariable adjusted mean difference = 0.48; 95% CI: 0.19, 0.77; P for trend = 0.001) (29).

##### White Matter Hyperintensities and Cerebral Infarcts

One study examined the presence of WMH and cerebral infarcts (37). While MeDi was not related to the presence of WMH, greater MeDi adherence was significantly associated with lower odds of cerebral MRI infarcts 5.8 years later (*OR* = 0.89; 95% CI = 0.80, 0.99; *P* = 0.04). This association was attenuated however, after exclusion of individuals with stroke (*n* = 86) and dementia (*n* = 46) (*OR* = 0.90; 95% CI = 0.80, 1.00; *P* = 0.07) (37).

#### Other Dietary Patterns and Structural Neuroimaging Markers

One study examined diet using the AHEI-2010 (33) and the other, PCA-derived prudent and western DPs (20) in relation to structural neuroimaging markers. Adherence to the AHEI-2010 was associated with increased total hippocampal volumes (HCV) measured 13 years later [β per each 1 SD increase in AHEI-2010 score (1 SD = 8.7 points) = 0.11; 95% CI: 0.02, 0.21]. Each 1 SD increase in AHEI-2010 score was associated with an increase of 92.5 mm^3^ (*SE* = 42 mm^3^) in total HCV, and when assessing each; an increase of 56.3 mm^3^ (*SE* = 23 mm^3^) in left HCV, and 36.2 mm^3^ (*SE* = 22.7 mm^3^) increase in right HCV (33). Similarly, better adherence to a prudent DP was significantly associated with larger left HCV, equal to 45.7 mm^3^ (S.E = 22.9 mm^3^). In contrast, a western DP was significantly associated with smaller left HCV, equal to 52.6 mm^3^ (S.E = 26.6 mm^3^). No associations were found between either *a posteriori* DP or right HCV (35). Change in left or right HCV across 4 years was also not significantly associated with the prudent DP (β_prudent×time_ = 20.8; *SE* = 24.4; *P* = 0.40) or western DP (β_western×time_ = 27.2; *SE* = 28.8; *P* = 0.34) (35).

#### Mediterranean Diet and Functional Neuroimaging Markers

Three studies assessed MeDi adherence in relation to functional neuroimaging markers, including brain glucose metabolism and Aβ load (30, 34, 39).

##### Brain Glucose Metabolism

Two studies used (18)F-fluorodeoxyglucose (FDG)-PET to assess glucose metabolism among middle-aged USA adults (34, 39). Low MeDi adherence was associated with significant reductions in the cerebral metabolic rate of glucose (CMRglc) specifically within the bilateral temporal cortex (*P* = 0.001) (34). Furthermore, the rate of CMRglc decline within both the temporal and posterior cingulate cortices among the group with low MeDi adherence was significantly faster than that of the high MeDi group (P interaction = 0.002). This equated to a decline of 3.83% (low MeDi group) (0.028 ± 0.049 units/year) compared to <1% (high MeDi group) (0.018 ± 0.054 SUVR units/year) per year from baseline (34). Similarly, Walters and colleagues also reported that lower adherence to the MeDi was significantly associated with a higher rate of FDG decline within the posterior cingulate cortex (β = 0.010; *SE* = 0.005; *P* = 0.043) but not in the frontal cortex (β = -0.012; *SE* = 0.007; *P* = 0.072) (39).

##### Amyloid Load

Three studies examined repeated measures of cerebral Aβ deposition up to 3 years (30, 34, 39). Two of the three studies found that decreased adherence to the MeDi was significantly associated with increased Aβ load among both middle-aged and older adults (30, 34). Lower MeDi adherence was associated with both higher Pittsburgh compound B (PiB) uptake at baseline and increased rate of PiB uptake across 3 years (*P* = 0.001) in middle-aged adults (34). Change in the low MeDi group was on average, 0.028 ± 0.031 SUVR units/year, equal to a 3% increase per year, compared to higher MeDi adherence which was on average, 0.009 ± 0.020 SUVR units/year, equal to a <1% increase per year (34). The Aβ accumulation rate within the AIBL cohort of older adults was higher than that of the cohort within the study by Berti and colleagues, at a rate of 0.050 SUVR units/year, meaning each 1-point increase in MeDi score was equal to 20% decrease in Aβ load per year (β = -0.01; S.E = 0.004; *P* = 0.0070) (30). However, it should be noted this cohort consisted entirely of individuals defined as Aβ accumulators by authors. In contrast, Walters and colleagues found no significant association between MeDi adherence and changes in Aβ in the frontal cortex among middle-aged adults (β = -0.016; *SE* = 0.011; *P* = 0.104) (39).

### Risk of Bias Assessment

An overview of the risk of bias assessment of all included studies is presented in Table 3. All studies included in the review were rated as medium (29, 30, 33, 35–39). Only one study achieved a high rating with a maximum total score of nine (34). The results of the quality assessment can be found in Table 3. Most studies were lacking within the selection domain, more specifically regarding ascertainment of exposure (due to most using self-reported assessment methods) and demonstration that the outcome was not present at baseline.

**TABLE 3:** Results of quality assessment of included studies using Newcastle-Ottawa Scale.

Authors, year	Representativeness of the exposed cohort	Selection of the non-exposed cohort	Ascertainment of exposure	Demonstration that outcome of interest was not present at baseline	Comparability of cohorts on the basis of the design or analysis	Assessment of outcome	Was follow-up long enough for outcomes to occur?	Adequacy of follow-up of cohorts	Overall rating
Akbaraly et al. (33)	★	★			★★	★	★	★	Medium
Berti et al. (34)	★	★	★	★	★★	★	★	★	High
Jacka et al. (35)	★	★		★	★★	★	★	★	Medium
Luciano et al. (36)	★	★			★★	★	★	★	Medium
Pelletier et al. (29)		★	★		★★	★	★	★	Medium
Rainey-Smith et al. (30)		★		★	★★	★	★	★	Medium
Scarmeas et al. (37)	★	★	★		★★	★	★	★	Medium
Walters et al. (39)	★	★		★	★★	★	★	★	Medium
Titova et al. (38)	★	★			★★	★	★	★	Medium

## Discussion

### Summary of Overall Findings

This systematic review evaluates results from nine prospective studies including approximately 2,540 individuals. No intervention evidence was evaluated, as no RCTs were eligible for inclusion. Consequently, it is difficult to understand the application of neuroimaging markers within future dietary interventions, as we are reliant on evidence gathered from prospective observational studies Within included studies, the MeDi was examined as the DP exposure in the majority (*n* = 7), with only two studies examining other DPs. Findings for associations between the MeDi and structural neuroimaging markers were mixed from the small number of studies available. MeDi was consistently not associated with a change in volumes of key brain regions such as GMV, WMV, or cortical thickness but was associated with reduced risk of cerebral infarcts and lower total brain atrophy. One study also found evidence of a protective association between MeDi and WMI (29). Other DPs (AHEI-2010 and PCA-derived DPs) were linked to higher HCV (33, 35) which is particularly relevant for AD, given that hippocampal atrophy is a major AD characteristic that often predates clinical diagnosis (42–44). HCV is considered one of the earliest structural MRI markers for AD, with studies suggesting atrophy rates may deviate from normal ageing as far as 3–5.5 years prior to diagnosis (45, 46). The MeDi appeared to be more consistently protective against hypometabolism (34, 39) and Aβ load (30, 34) from mid-life to older age, which preceded any evidence of cognitive impairment. Such results are important, given that impairments in systemic metabolism (such as hypometabolism) and Aβ accumulation are pathophysiologic hallmarks of AD (47–49). However, current data is unable to discern the exact mechanisms behind these associations; specifically, whether a healthy DP helps by inhibiting Aβ accumulation/deposition, by improving Aβ clearance, or through both (30). Data in this area are currently limited, with few prospective studies examining Aβ load in relation to DPs among humans (30, 34, 39) consequently warranting further investigation.

Overall, this systematic review of available prospective data suggests a protective association between healthy DPs and neuroimaging markers and confirms findings from a prior review involving prospective studies to 2017 (28) and other reviews involving both prospective and cross-sectional study evidence (25–27). The exact mechanisms responsible for how healthy DPs may be neuroprotective are not known but high-quality DPs such as the MeDi could improve vasculature functioning by reducing oxidative stress and inflammation. Better adherence to a healthy diet such as the MeDi, may serve to decrease the likelihood of vascular comorbidities including hypertension, metabolic syndrome, dyslipidaemia, and cardiopathy (50). It is proposed this subsequently promotes healthy brain ageing by reducing an individuals’ risk of vascular-related brain pathologies. The main drivers of vascular preservation likely involve enhanced endothelial capacity, healthy cerebral blood flow and reduced inflammation and oxidative stress. The neuroimaging markers commonly associated with vascular pathologies relate to the potential progression of small vessel disease, such as the volume and enlargement of WMH, lesions, infarcts, and preceding this, loss of WMI (51). Previous cross-sectional studies have reported associations between greater MeDi adherence and reduced volume of WMH (52). A main predictor of WMH volume within this relationship, was suggested to be the ratio of monounsaturated fats to saturated fats within the MeDi (52). Greater fish consumption, as recommended by the MeDi, has also been independently related to reduced WMH volume and increased brain volumes (53–56). The vascular pathway is supported by findings of this review, as two studies found associations greater adherence to the MeDi and reduced appearance of vascular-related neuroimaging markers (specifically reduced odds of cerebral infarcts, and improved white matter tract integrity) (34, 37).

Loss of white matter microstructural integrity is also proposed to reflect both myelin breakdown and axonal damage (57, 58). The neurodegeneration hypothesis proposes pathological brain changes such as structural atrophy, formation, deposition of Aβ plaques and oxidative stress occur due to a combination of events. This includes axonal cytoskeleton degeneration, myelin degradation and an accumulation of reactive oxygen species. Findings from six studies included within this review lend support to a healthy DP for supporting axonal maintenance and protecting against neurodegeneration. For example, through reduced hippocampal and total brain atrophy over time (33, 35, 36), decreased Aβ deposition (30, 34) and slower declines in glucose metabolism within specific regions (34, 39). Data from several cross-sectional studies have also shown protective associations of healthy DPs on neuroimaging outcomes (22, 53, 59–62).

Healthy DPs such as the MeDi, AHEI-2010 and prudent DP derived a posteriori share similarities including high consumption of foods rich in polyphenols and antioxidants such as fruits/vegetables. Such compounds may exert protective effects by obstructing neuronal oxidation, maintaining cellular homeostasis and hence, limit abnormal intracellular responses such as senescence and alterations within brain plasticity (63–66). Furthermore, healthy DPs such as the MeDi, DASH and AHEI-2010 are suggested to reduce levels of inflammatory macrophage proteins, cytokines, and chemokines (67–70). Neuroinflammation plays a major role in both oxidative and neurodegenerative pathways, thus review outcomes support the role of healthy DPs such as the MeDi, AHEI-2010 and prudent DPs in decreasing neuroinflammation. In support of this, one study also found a western DP, often associated with systemic inflammation, was associated with decreased HCV (35).

### Inconsistencies Within Study Methodology

This review also serves to highlight several inconsistencies within the designs of included studies when investigating associations between DPs and neuroimaging markers. This includes robustness of study design, potential biases within populations assessed, and measurement of neuroimaging markers, dietary intake and consequent DPs as discussed below.

#### Study Design

All studies included in this review were prospective cohort studies. Most studies were rated as medium quality, with the exception of one rated as high. None of the included studies were assessed as poor quality. Although the design of such prospective studies has several strengths, they cannot determine causality and may be biased toward residual confounding. All studies controlled for age and sex. Most studies controlled for important confounders that influence both diet and neurocognition, as eight (29, 30, 33–39) controlled for education, six (29, 30, 34, 36, 37, 39) controlled for APOE E4 status, and eight (29, 33–39) controlled for ≥1 measure of cardiovascular risk (i.e., diabetes, hypertension). However, only two studies controlled for socioeconomic status (SES) (33, 35). This is interesting, given the association between SES and cognition across the lifespan (71), in addition to the link between SES and diet (72). Although both studies which controlled for SES in this review found statistically significant findings to suggest an influence of DP on neuroimaging measures, future studies may wish to consider controlling for this factor within analyses. Finally, no included studies were RCTs. Subsequently, this means it is difficult to draw causal inferences from this review.

#### Populations Assessed

No study focused exclusively on an age category and therefore each included older adult populations (aged ≥ 60). Due to this, combined with conflicting findings overall it is difficult to draw conclusions as to whether associations may differ dependent upon the life stage. However, from the two studies that did include younger adult individuals (age ≥ 30), both found significant associations between MeDi and structural (specifically WMI) and functional neuroimaging markers (specifically Aβ and glucose metabolism) (34, 39). It has been suggested that the earliest pathological changes may be detected using neuroimaging markers between 15 and 30 years preceding symptom onset (42, 73, 74). As most studies captured older-aged individuals, it is difficult to understand how long an individual must have adhered to a DP, to influence changes in brain ageing. The majority of studies were conducted among educated populations and only four studies reported ethnic status (mostly white populations except for WHICAP). Consequently, more studies are required in diverse populations, particularly those at higher risk of AD in later life.

#### Dietary Assessment

The limitations associated with methods of dietary intake measurement, specifically FFQs as the most frequently used method of assessment have previously been acknowledged (75, 76). This includes potential misreporting from the participant due to both recall bias and social desirability bias; as “healthy” foods are likely to be reported more frequently in comparison to “unhealthy” foods. Based on this, studies using FFQs to measure diet intake should aim to validate the obtained data to ensure a robust assessment of dietary intake using an alternative methodology such as combined measurement of various recovery-based nutrient biomarkers (e.g., doubly labelled water for energy intake or urinary nitrogen for protein intake) or another quantitative method as reference, such as food records or 24 h diet recalls (77, 78).

To validate subjective methods should be considered within studies to ensure a robust assessment of dietary intake. Furthermore, most studies within this review also failed to assess diet at multiple time points and relied on FFQ reporting from years prior to neuroimaging marker measurement. Older adulthood has previously been suggested to be a period where individuals may experience reduced diet stability (79–81). Therefore, it is difficult to rule out the risk of reverse causation within associations observed. Future studies should be conducted in earlier life, to understand how DPs may influence changes in neuroimaging markers across the adult life span.

#### Dietary Patterns Assessed

Most prospective studies to date have examined associations between the MeDi and neuroimaging markers, which can help elucidate potential neuroprotective mechanisms. However, differences were noted between the applications of the MeDi scoring system within the included studies. Although all studies applied a similar scoring index, there were interindividual study differences in food components, specific cut-offs for components (i.e., alcohol), and adjustments for energy intake. For example, Titova et al. (38) combined vegetables and legumes into one component, added potatoes to the cereals component, and replaced monounsaturated fats with polyunsaturated fats in the fats ratio component. Differences were also noted between studies in the scoring cut-offs for the alcohol component. Adaptations such as the above may contribute further towards inconsistent findings within this area; as it becomes difficult to understand if observed associations are due to the synergy within the MeDi pattern or due to its individual food components, which often differ between studies. Similarly, the AHEI-2010 score applied in one study within this review (33) was also adapted from the original score index by decreasing the recommended intake of alcohol, based on the evidence within the research topic. Furthermore, as previous studies focus on healthy DPs, there is a substantial lack of studies exploring the effects of unhealthy DPs on neuroimaging markers. Previous animal studies have illustrated that a western diet is associated with reduced cognitive functioning, due to reduced neuronal plasticity and higher levels of inflammation and oxidative stress (82, 83). This review found only one longitudinal study that investigated an unhealthy DP in relation to neuroimaging markers in humans. Further research should therefore address this gap and aim to understand the influence and mechanisms of poor-quality DPs on brain ageing, using neuroimaging markers. This would increase understanding of their effects on wider populations, such as those across westernised countries who often consume such diets.

#### Neuroimaging Marker Measurement

Only five studies assessed change in neuroimaging markers at two or more time points. The maximum length of time in any study between these two points was 4 years. Therefore, it is difficult to interpret to distinguish between normal vs. pathological brain changes and how this may associate with any given DP. The main modality of neuroimaging assessment across studies was via MRI (8 studies), with only three studies using other methodologies (i.e., PET and DTI) (30, 34, 39). However, it has been suggested that in pathological brain ageing, changes in Aβ deposition may occur first, followed by neurodegenerative changes such as Tau burden and structural brain atrophy (42, 84). Hence, this may help to explain why four (29, 30, 34, 39) of the eight studies in cognitively healthy adults found significant associations between adherence towards a DP and neuroimaging markers other than structural MRI. Another noteworthy finding from this review was the substantial lack of studies that assessed important sub-cortical brain regions such as the hippocampus that are vulnerable to AD. Previous animal studies have also suggested diet may impact hippocampal neurogenesis, by demonstrating a damaging role of saturated fats and refined sugar on hippocampal neurogenesis (85, 86). Interestingly, only two studies (33, 35) in this review investigated hippocampal volume. It would therefore be beneficial for future studies to adopt a multi-modal imaging approach; as this would enable us to understand the earliest and optimal window for nutritional interventions to support healthy brain ageing. The use of both modalities in combination with neuropsychological testing would also provide a comprehensive overview of structural and functional brain changes; how these correlate with cognitive performance and potential associations between DPs and the food components within that DP. Finally, it must be noted that this systematic review may be prone to bias as articles published in languages other than English were excluded. As a meta-analysis was not possible, it is also difficult to determine the extent of publication bias within the studies included.

## Conclusion

This systematic review of prospective data suggests a protective association between healthy DPs and neuroimaging markers and agrees with prior reviews involving cross-sectional studies (25–27). Evidence, while limited, suggests healthy DP’s, such as the MeDi or AHEI-2010 may exert beneficial changes on both brain structure (reduced brain atrophy) and function (maintenance of glucose metabolism and reduced Aβ accumulation). Although the mechanisms underpinning these associations require further elucidation, both vascular and neurodegenerative processes are likely involved. This review highlighted the value of different neuroimaging techniques (i.e., MRI and PET) and markers in detecting and capturing subtle changes within the brain, which often occur prior to cognitive impairment. No RCTs were available for inclusion in this review and highlights a clear gap within the research field. Future intervention studies in cognitively healthy adults that include multi-modal neuroimaging measures would advance current knowledge on whether adopting a healthy diet impacts brain health. Undertaking such studies may be especially relevant and beneficial in reducing the likelihood of dementia among high-risk population groups, such as those with diabetes. Furthermore, future studies should be designed to adopt a life-span approach, conducting rigorous follow-up reviews with multi-modal neuroimaging techniques and validated dietary intake assessment at regular time points. This will enable a comprehensive depiction of how the length of adherence towards a specific DP may alter an individuals’ brain ageing trajectory. Finally, although healthy DPs may offer neuroprotective benefits, the exact combination of foods and nutrients providing such benefits remain unknown. Consequently, future studies should also seek to address the paucity of research investigating DPs other than the MeDi.

## Author Contributions

RT, CM, and JW conceived and designed the study. RT conducted the search. RT and CM completed screening and extraction processes with any disagreements discussed in the presence of JW, RT, and RO’N and performed quality assessment. RT, CM, JW, RO’N, and FP contributed to drafting and completing the manuscript. All authors contributed to the article and approved the submitted version.

## Conflict of Interest

The authors declare that the research was conducted in the absence of any commercial or financial relationships that could be construed as a potential conflict of interest.

## Publisher’s Note

All claims expressed in this article are solely those of the authors and do not necessarily represent those of their affiliated organizations, or those of the publisher, the editors and the reviewers. Any product that may be evaluated in this article, or claim that may be made by its manufacturer, is not guaranteed or endorsed by the publisher.

## Abbreviations

AD: Alzheimer’s Disease
AHEI-2010: Alternate Healthy Eating Index-2010
Aβ: Amyloid-β
Cmrglc: Cerebral Metabolic Rate Of Glucose
DP: Dietary pattern
DTI: Diffusion Tensor Imaging
EVOO: Extra Virgin Olive Oil
FDG-PET: (18)F-Fluorodeoxyglucose Positron Emission Tomography
FFQ: Food frequency questionnaire
GMV: Grey Matter Volume
HCV: Hippocampal Volume
MeDi: Mediterranean diet
MMSE: Mini-Mental State Examination
MRI: Magnetic Resonance Imaging
NOS: Newcastle Ottawa Scale
PCA: Principal Component Analysis
PET: Positron Emission Tomography
PiB: Pittsburgh compound B
PRISMA: Preferred Reporting Items for Systematic Reviews and Meta-Analyses
PROSPERO: International Prospective Register of Systematic Reviews
RCT: Randomised Controlled Trial
SUVR: Standard Uptake Volume Ratio
TBV: Total Brain Volume
WMI: White Matter Integrity
WMV: White Matter Volume.
