# Supplementary material for: Associations Between Dietary Patterns and Neuroimaging Markers: A Systematic Review
Source: Front Nutr. 2022 Apr 26;9:806006. doi: 10.3389/fnut.2022.806006 (PMC9097077; doi:10.3389/fnut.2022.806006)
Supplement: Supplementary file 1 [file Table_1.DOCX]

**Supplementary Table 1. Search strategy for systematic review.**

| **NUMBER** | **SEARCH TERM** |
| --- | --- |
|  | “Dietary pattern*”.mp. |
|  | “Dietary habit*”.mp. |
|  | “Nutrient pattern*”.mp. |
|  | Diet therapy/ or caloric restriction/ or diet, carbohydrate loading/ or diet, diabetic/ or diet, carbohydrate-restricted/ or diet, high-protein low-carbohydrate/ or diet, ketogenic/ or diet, fat-restricted/ or diet, gluten-free/ or diet, high-protein/ or diet, mediterranean/ or diet, paleolithic/ or diet, protein-restricted/ or diet, reducing/ or diet, sodium-restricted/ or diet, vegetarian/ or diet, macrobiotic/ or diet, vegan/ or dietary approaches to stop hypertension/ |
|  | Diet, high-fat/ or diet, western/ or diet, healthy/ |
|  | “DASH diet*".mp. |
|  | “Dietary Approaches to Stop Hypertension*".mp. |
|  | “MIND Diet*".mp |
|  | "Mediterranean-DASH Intervention for Neurodegenerative Delay*".mp. |
|  | "Mediterranean Diet*".mp. |
|  | "Low-GI Diet*".mp. |
|  | "Low-Fat Diet*".mp. |
|  | “Low-Calorie Diet*".mp. |
|  | “Healthy Diet*".mp. |
|  | “Prudent Diet*".mp |
|  | “Healthy Eating Index Diet*".mp. |
|  | “Healthy Eating Index*".mp. |
|  | “Alternative Healthy Eating Index Diet*".mp. |
|  | “Alternative Healthy Eating Index*".mp. |
|  | “Inflammatory diet*".mp. |
|  | “Diet* Inflammatory Index".mp. |
|  | "Food pattern*".mp |
|  | “Diet”.ti. |
|  | exp Cognition/ or cognition.mp. |
|  | exp Cognition Disorders/ or "Cognitive Disorder*".mp. |
|  | exp Neuropsychological Tests/ or "Neuropsychological Test*".mp |
|  | exp Psychological Tests/ |
|  | “Psychological Test*".mp. |
|  | exp Memory, Long-Term/ or Memory.mp. or exp Memory, Short-Term/ or exp "Memory and Learning Tests"/ or exp Spatial Memory/ or exp Memory Disorders/ or Memory/ or exp Memory, Episodic/ |
|  | Dementia*.mp. or exp Dementia/ or exp Frontotemporal Dementia/ or exp Dementia, Vascular/ or exp Dementia, Multi-Infarct/ or exp "Mental Status and Dementia Tests”/ |
|  | “cognitive function*".mp. |
|  | “Cognitive impairment*".mp. |
|  | “Cognitive Decline*".mp. |
|  | “Mild Cognitive Impairment*".mp. |
|  | “Cognitive Ageing".mp. |
|  | exp Cognitive Aging/ |
|  | exp Aging/ or "Ageing".mp. |
|  | “Age-Associated Cognitive Decline".mp. |
|  | “Memory Impairment".mp |
|  | “Brain Ag*".mp. |
|  | exp Neuroimaging/ or exp Functional Neuroimaging/ or Neuroimaging.mp |
|  | “Magnetic Resonance Imaging".mp. or exp Magnetic Resonance Imaging/ |
|  | “Functional Magnetic Resonance Imaging".mp. |
|  | exp Electroencephalography Phase Synchronization/ or exp Electroencephalography/ or Electroencephalography.mp. |
|  | Magnetoencephalography.mp. or exp Magnetoencephalography/ |
|  | “Tomography, X-Ray Computed".mp. or exp Tomography, X-Ray Computed/ |
|  | “Positron-Emission Tomography".mp. or exp Positron-Emission Tomography/ |
|  | exp Spectroscopy, Near-Infrared/ or "Functional Near-Infrared Spectroscopy".mp. |
|  | “Diffusion Tensor Imaging".mp. or exp Diffusion Tensor Imaging/ |
|  | “Brain Structure".mp. |
|  | “Brain Function*".mp. |
|  | “Brain Morphology*".mp. |
|  | “Brain Imaging*".mp. |
|  | “MRI”.mp. |
|  | “PET”.mp. |
|  | “CT”.mp. |
|  | “MEG”.mp. |
|  | “fNIRS”.mp. |
|  | “DTI”.mp. |
|  | “SPECT”.mp. |
|  | “EEG”.mp. |
|  | 1 or 2 or 3 or 4 or 5 or 6 or 7 or 8 or 9 or 10 or 11 or 12 or 13 or 14 or 15 or 16 or 17 or 18 or 19 or 20 or 21 or 22 or 23 |
|  | 24 or 25 or 26 or 27 or 28 or 29 or 30 or 31 or 32 or 33 or 34 or 35 or 36 or 37 or 38 or 39 or 40 or 41 or 42 or 43 or 44 or 45 or 46 or 47 or 48 or 49 or 50 or 51 or 52 or 53 or 54 or 55 or 56 or 57 or 58 or 59 or 60 or 61 |
|  | 62 and 63 |
|  | Limit 64 to (english language and humans) |
|  | Limit 65 to “all adult (19 plus years)” |
