# Supplementary material for: Associations Between Dietary Patterns and Neuroimaging Markers: A Systematic Review
Source: Front Nutr. 2022 Apr 26;9:806006. doi: 10.3389/fnut.2022.806006 (PMC9097077; doi:10.3389/fnut.2022.806006)
Supplement: Supplementary file 2 [file Table_2.docx]

| **Supplementary Table 2. An overview of the DP’s studied within all included studies.** | | | |
| --- | --- | --- | --- |
| **Authors, year, *study name*, country** | **DP studied:** | **Scoring system used  (if a-priori):** | **Food components included or characteristics of a-posteriori pattern:** |
| Pelletier et al., (2015); *Three City Study;* France. | MeDi | Trichopoulou et al., (2003) | 1. Fruits  2. Vegetables 3. Legumes 4. Cereals 5. Fish 6. Dairy 7. Meat  8. Monounsaturated fat to saturated fat ratio  9. Mild alcohol intake |
| Scarmeas et al., (2011) *WHICAP Study;* USA. | MeDi | Trichopoulou et al., (2003) | 1. Fruits  2. Vegetables 3. Legumes 4. Cereals 5. Fish 6. Dairy 7. Meat  8. Monounsaturated fat to saturated fat ratio  9. Mild alcohol intake |
| Titova et al., (2013); *Prospective Investigation of the Vasculature in Uppsala Seniors Cohort;* Sweden. | MeDi | Trichopoulou et al., (2003) | 1. Fruits  2. Vegetables/Legumes 3. Cereals and Potatoes 4. Fish 5. Dairy 6. Meat  7. Polyunsaturated fat to saturated fat ratio  8. Mild alcohol intake |
| Berti et al., (2018); USA. | MeDi | Trichopoulou et al., (2003) | 1. Fruits  2. Vegetables 3. Legumes 4. Cereals 5. Fish 6. Dairy 7. Meat  8. Monounsaturated fat to saturated fat ratio  9. Mild alcohol intake |
| Walters et al., (2018); USA. | MeDi | Trichopoulou et al., (2003) | 1. Fruits  2. Vegetables 3. Legumes 4. Cereals 5. Fish 6. Dairy 7. Meat  8. Monounsaturated fat to saturated fat ratio  9. Mild alcohol intake |
| Luciano et al., (2017);  *Lothian Birth Cohort;* Scotland, UK. | MeDi | Trichopoulou et al., (2003) | 1. Fruits  2. Vegetables 3. Legumes 4. Cereals 5. Fish 6. Dairy 7. Meat  8. Monounsaturated fat to saturated fat ratio  9. Mild alcohol intake |
| Rainey-Smith et al., (2018), *Australian Imaging, Biomarkers and Lifestyle Study of Ageing*; Australia. | MeDi | Trichopoulou et al., (2003) | 1. Fruits  2. Vegetables 3. Legumes 4. Cereals 5. Fish 6. Dairy 7. Meat  8. Monounsaturated fat to saturated fat ratio  9. Mild alcohol intake |
| Akbaraly et al., (2018);  *Whitehall II Imaging Sub-study;* UK. | AHEI-2010 | Chiuve et al., (2012). | 1. Fruits 2. Vegetables 3. Nuts and legumes 4. Sodium 5. Red and processed meat  6. Wholegrains 7. Sugar sweetened beverages and fruit juice 8. Long chain omega-3 fats 9. Polyunsaturated fatty acids (does not include Omega-3 PUFA). 10. Trans fat  11. Alcohol intake |
| Jacka et al., 2015,  *PATH Sub-study;* Australia. | A-posteriori derived (prudent and western) | N/A. | **1. Prudent** Characterised by high consumption of fresh vegetables, salad, fruit & grilled fish.  **2. Western** Characterised by high consumption of roast meat, sausages, hamburgers, steak, chips, crisps and soft drinks. |

Reference List:

Trichopoulou A, Costacou T, Bamia C, Trichopoulos D. Adherence to a Mediterranean diet and survival in a Greek population. N Engl J Med (2003) 348(26):2599-608. Epub 2003/06/27. doi: 10.1056/NEJMoa025039. PubMed PMID: 12826634.

Chiuve SE, Fung TT, Rimm EB, Hu FB, McCullough ML, Wang M, et al. Alternative Dietary Indices Both Strongly Predict Risk of Chronic Disease. The Journal of Nutrition (2012) 142(6):1009-18. doi: 10.3945/jn.111.157222.
